# Intermittent colonic exoperistalsis for chronic constipation in spinal cord-injured individuals. A long-term structured patient feedback survey to evaluate home care use

**DOI:** 10.1038/s41394-023-00597-z

**Published:** 2023-07-29

**Authors:** Jana Bremer, Jörn Bremer, Maike König, Peter Koßmehl, Ines Kurze, Jeannette Obereisenbuchner, Elisabeth Weinschenk, Immaculada Herrero-Fresneda

**Affiliations:** 1 BDH-Klinik Greifswald, Greifswald, Germany; 2 Zentralklinik Bad Berka, Bad Berka, Germany; 3 Neurologische Fachkliniken Beelitz-Heilstätten, Beelitz, Germany; 4 Scientific and Medical Department, usMIMA S.L, Barcelona, Spain

**Keywords:** Quality of life, Spinal cord diseases, Outcomes research, Functional gastrointestinal disorders

## Abstract

**Study design:**

Structured patient feedback survey evaluating real-world home care use.

**Objectives:**

To assess the long-term effectiveness, tolerability, and satisfaction with the intermittent colonic exoperistalsis (ICE) treatment device MOWOOT in spinal cord-injured (SCI) individuals with chronic constipation.

**Setting:**

Four specialized German hospitals.

**Methods:**

SCI individuals with chronic constipation were invited to use MOWOOT 10–20 min daily and answer a questionnaire about their bowel situation before treatment (feedback 1, F1) and after ≥10 months of use (feedback 2, F2). Collected variables were device use, bowel function effectiveness, chronic constipation symptoms, concomitant use of laxatives and evacuation aids, and satisfaction with bowel function and management, which were compared between time points. At F2, participants reported efficacy, tolerability/side effects, and ease of use.

**Results:**

Eleven participants used the device for a mean (SD) of 13.27 (4.03) months. From F1 to F2, mean time per evacuation decreased by 24.5 min (*p* = 0.0076) and the number of failed attempts to evacuate/week, by 1.05 (*p* = 0.0354) with a tendency toward increased bowel movements and softer stool consistency, and decreased incomplete bowel movements. Participants experienced decreased difficulty/strain (*p* = 0.0055), abdominal pain (*p* = 0.0230), bloating (*p* = 0.0010), abdominal cramps (*p* = 0.0019), and spasms (*p* = 0.0198), without significant changes in the use of laxatives and evacuation aids. Satisfaction with bowel function and management improved (*p* = 0.0095) and more participants reported being very satisfied/satisfied (*p* = 0.0300). Most reported tolerability, efficacy, and ease of use as very good/good.

**Conclusion:**

Long-term in-home ICE treatment improved bowel function and chronic constipation symptoms in SCI individuals, providing clinical benefits to this population.

**Sponsorship (MOWOOT devices lending):**

4 M Medical GmbH, Norderstedt, Germany.

## Introduction

Spinal cord injury (SCI) results in the loss of motor, sensory, and autonomic functions, although symptoms vary depending on the severity and affected area [1]. Among the autonomic functions affected, the lack of motility alters gastrointestinal activities, resulting in impaired ability to store and evacuate the feces. Together with the motor and sensory functions, autonomous defects impacting bowel function impair the quality of life (QoL) of SCI individuals, affecting their mental health and social life [1–3]. For this reason, it is essential to implement a management plan to improve the symptoms of bowel dysfunction.

Chronic constipation affects up to 80% of SCI individuals and is associated with multiple impairments, such as incontinence, fecal impaction, and hemorrhoids [4]. Current guidelines include a series of sequential approaches to managing chronic constipation [5]. First, lifestyle changes, such as increased fluid intake or exercise, followed by pharmaceutical solutions. However, regular exercise may be challenging for SCI individuals, and pharmaceutical solutions, such as laxatives or suppositories, have shown limited efficacy [6, 7]. The last option is the invasive interventions, ranging from the minimally invasive enemas and transanal irrigation (TAI), to surgical procedures [8, 9].

Manual colon-specific massage applied by medical professionals (e.g., physiotherapists) is a non-invasive method with proven efficacy to treat chronic constipation [5, 10–12]. However, to be effective, the massage should be administered daily, making it expensive and with limited access and feasibility in the home setting. A solution to overcome these obstacles is the use of a mechanical device that can be self-administered and applies sequential pressure alongside the colon. MOWOOT is an automated intermittent colonic exoperistalsis (ICE) treatment device simulating the natural peristaltic contractions of the colon. In a multicentric clinical trial, the ICE device has shown safety and efficacy in patients with chronic constipation due to several causes, reducing constipation symptoms and evacuation aids, with a concomitant increase in the number of complete bowel movements and excellent adherence [13].

Despite the clinical benefits of ICE device treatment and the advantages of its autonomous use, studies focused on SCI individuals are missing. Furthermore, assessing the feasibility of management strategies that promote individuals’ autonomy in the home setting, such as ICE device use, is important to obtain valuable real-world data. The aim of this structured patient feedback survey was to assess the long-term effectiveness and tolerability, as well as the satisfaction with the ICE device in a home setting in SCI individuals diagnosed with chronic constipation.

## Methods

### Survey design and participants

This was a structured patient feedback survey evaluating the home care use of the ICE medical device (MOWOOT) to treat chronic constipation due to SCI. Participants were recruited from February 2019 to September 2020 in four specialized hospitals in Germany. Eleven SCI individuals with chronic constipation and slow intestinal transit were selected by their physicians and invited to use the ICE device at their homes and join this evaluation. Constipation was diagnosed during a regular visit to the SCI center as part of lifelong follow-up care and based on self-reported symptoms (unsatisfactory defecation characterized by infrequent stool, difficult stool passage, or both for at least the previous three months) [14]. No additional selection criteria were considered and therefore, participants mainly had neurogenic bowel dysfunction (NBD)-related chronic constipation, which may be combined with other causes of chronic constipation, including side effects of medications and complications due to SCI concomitant dysfunctions. Those who accepted were provided with a MOWOOT device and were asked to give a voluntary, anonymous structured feedback. 4 M Medical GmbH supplied the ICE device free of charge to the participants as a supplement to their current treatment. Participants were instructed to perform the ICE treatment for 10–20 min daily.

During treatment, they were free to adapt the supportive medication (e.g., laxatives or suppositories) or device settings (massage duration and level) at any time, and they were also asked to maintain their usual lifestyle and diet habits. In this regard, following a moderately active lifestyle and a healthy diet including fiber and enough fluid intake is the first step in bowel management, and all participants with chronic constipation are supposed to do it. Participant feedback was collected using a self-administered questionnaire designed to collect data at baseline (F1) and after at least ten months of ICE device use (F2). It was sent to all participants simultaneously, regardless of their treatment start date. Therefore, the period of device use evaluated was different for each participant. This structured participant feedback was anonymous, i.e., it followed all confidentiality requirements for personal data protection in compliance with European legislation (Supplementary materials).

### Study device: MOWOOT-II system

**Fig. 1:**
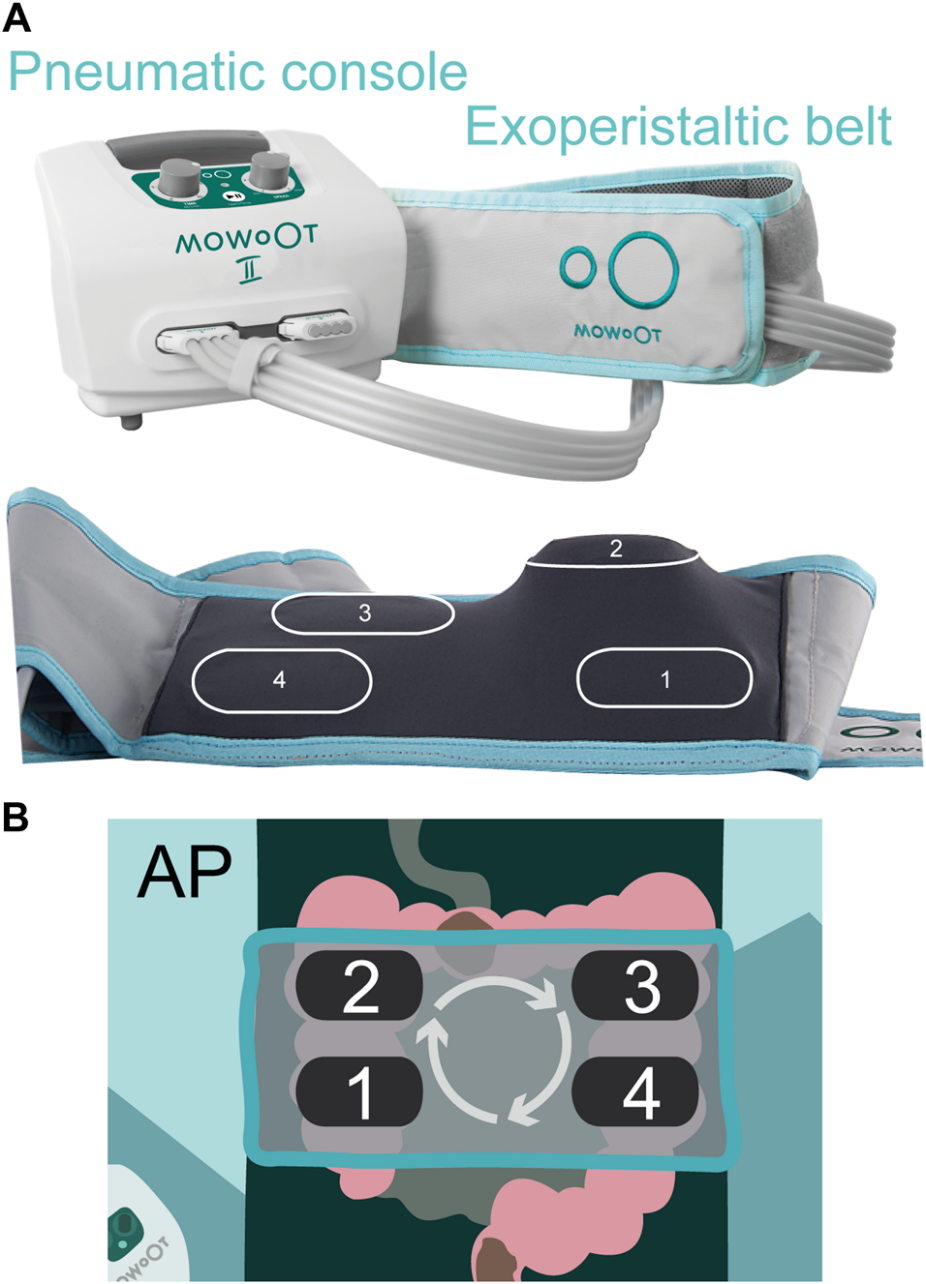
MOWOOT-II intermittent exoperistalsis device. Image of the MOWOOT-II device with the pneumatic console and the exoperistaltic belt. The inner side of the exoperistaltic belt is shown below (**A**). Diagram depicting the inflating-deflating sequence of the four pneumatic active elements (**B**).

The MOWOOT-II system (usMIMA S.L., Barcelona, Spain) is a CE-certified medical device class IIa that automatically gives an intermittent colonic exoperistalsis (ICE) treatment, simulating the natural peristaltic contractions of the colon. It is composed of two pieces: a pneumatic desktop device and an exoperistaltic belt (Fig. 1). The desktop device contains the control panel and provides an energy source, while the pneumatic active elements of the belt inflate and deflate consecutively in a clockwise direction over the ascending and descending colon.

### Variables and assessments

The questionnaire, which was designed for the purpose of this study, included a series of items to report demographic data (i.e., age and sex) and feedback regarding device use, effectiveness, including bowel function management and symptoms, concomitant use of laxatives and evacuation aids, tolerability, safety, and satisfaction. A detailed description of the variables collected, their quantification (i.e., semiquantitative), and transformation into categorical variables are provided as Supplementary methods.

### Statistical analysis

All analyses were performed using the intention–to-treat (ITT) population. Additionally, to investigate the effects of time of ICE device use, participants were categorized according to time under treatment (i.e., total period of device use) as <12 months and ≥12 months. Quantitative variables were described as the mean and standard deviation (SD). Paired measures were analyzed using the Student’s t-test, the Wilcoxon test, odd ratios, and the Chi-squared/Fisher’s exact test; comparisons between groups were performed using the two-way ANOVA. The statistical analysis is further described in the Supplementary methods. Statistical significance was set at a two-tailed *p*-value < 0.0500. Data processing and analyses were performed using GraphPad Prism software.

## Results

### Characteristics of survey participants and device use

The survey included 11 SCI individuals with chronic constipation; over half (63.6%) were men (Table 1). Participants were aged 40−49 years (*n* = 3), 50−59 years (*n* = 1), 60−69 years (*n* = 2), and >70 years (*n* = 5). The total period of ICE device use varied between participants (Table 1). Four participants used the device for <12 months and seven used it for ≥12 months.

**Table 1:** Characteristics of study participants and time under ICE treatment.

	Treatment < 12 m *n* = 4	Treatment ≥ 12 m *n* = 7	All *n* = 11
Demographic characteristics			
Sex			
Male, *n*	2	5	7
Female, *n*	2	2	4
ICE treatment			
Total period of ICE device use (months)			
* Mean (SD)*	10.50 (0.58)	14.86 (4.38)	13.27 (4.03)
* Min, max*	10, 11	12, 24	10, 24
Time of ICE device use			
Sessions/day, *mean (SD)*	1.15 (0.60)	1.37 (0.61)	1.29 (0.58)
Min/session, *mean (SD)*	18.75 (2.50)	19.29 (5.35)	19.09 (4.37)
Min/day, *mean (SD)*	21.75 (12.61)	26.14 (13.00)	24.54 (12.41)
Adherence (%)			
* Mean (SD)*	109 (63)	131 (65)	123 (62)
* Median (min, max)*	87.5 (60,200)	90 (75,200)	90 (60,200)

*ICE* intermittent colonic exoperistalsis; *SD* standard deviation.

Changes in diet and fluid intake between groups lacked statistical significance ( < 12 months vs. ≥12 months, Chi-squared/Fisher’s exact test, *p* = 0.4909). At F2, three out of the seven participants with ≥12 months of device use reported changes in lifestyle, consisting of a higher fiber intake (*n* = 1), a fluid intake of 1.5 L−2 L per day (*n* = 1), and a healthier diet (*n* = 1). The rest of participants reported no major changes.

At F2, participants were asked about the number of daily sessions and session duration (the instructed use was at least once a day for 10–20 min). Four of them used it for 40 min/day in two 20-min sessions, and four participants used it for 15 min/day in a single daily session. Two participants used it less than once a day in sessions of 20 min (*n* = 1) and >20 min (*n* = 1). Only one participant used it as proposed (20 min/day in one session). The mean time of use/day was slightly higher than recommended, resulting in a mean treatment adherence >100% (Table 1). There was no difference between participants followed up for <12 months and ≥12 months (two-way ANOVA, *p* = 0.5532 for min/day and *p* = 0.5299 for adherence).

### Effectiveness of ICE device use

Table 2 summarizes the variables associated with bowel function at F1 and F2. Participants reported decreased “time per evacuation”, decreased number of “failed attempts to evacuate” and decreased number of “incomplete movements”, and a concomitant increase in the number of “bowel movements”, indicating improved bowel function after treatment. Differences in “time per evacuation” and “failed attempts to evacuate/week” before and after months of treatment reached statistical significance. Average Bristol scores increased, although not significantly, indicating softer stool consistency, reaching mean scores ≤5. Subgroup analyses according to time under treatment showed a similar trend, especially in the subgroup of participants using the ICE device ≥12 months. In these participants, changes seemed more prominent than in the subgroup of participants who used the ICE device for <12 months, although without statistically significant differences.

**Table 2:** Variables associated with changes in bowel function and symptoms before (F1) and at ICE treatment (F2), *N* = 11.

	Months of use	n	F1	F2	F2 - F1	*P-*value
Bowel function						
Number of bowel movements/week^**a**^, *mean (SD)*	<12 m	4	3.62 (0.25)	4.12 (0.95)	0.50	0.3910
	≥12 m	6	1.75 (0.93)	4.36 (2.66)	2.17	0.1348
	All	10	2.50 (1.20)	4.27 (2.13)	1.50	0.0848
Number of failed attempts to evacuate/week^**a**^, *mean (SD)*	<12 m	4	1.25 (1.50)	0.50 (0.58)	−0.75	0.2152
	≥12 m	6	1.86 (1.31)	1.67 (0.82)	−1.25	0.1147
	All	10	1.64 (1.34)	0.60 (0.70)	−1.05	0.0354
Number of incomplete bowel movements/week^**a**^, *mean (SD)*	<12 m	3	2.67 (1.89)	1.67 (1.04)	−1.00	0.2254
	≥12 m	6	2.79 (4.20)	1.33 (1.75)	−1.67	0.2586
	All	9	2.75 (3.54)	1.44 (1.49)	−1.44	0.1346
Time per evacuation (min)^**a**^*, mean (SD)*	<12 m	4	83.75 (47.50)	63.75 (39.45)	−20.00	0.0663
	≥12 m	6	69.29 (27.45)	47.50 (18.91)	−27.50	0.0604
	All	10	74.55 (34.38)	54.00 (28.07)	−24.50	0.0076
Average Bristol score (1−7)^b^, *mean (SD)*	<12 m	2	4.50 (0.71)	5.00 (0.00)	0.50	1.0000
	≥12 m	7	2.86 (1.46)	4.57 (1.27)	1.71	0.1344
	All	9	3.22 (1.48)	4.67 (1.12)	1.44	0.0890
Symptoms						
Difficulty/strain (1−6)^b^, *mean (SD)*	<12 m	4	4.25 (2.87)	2.50 (1.91)	−1.75	0.2500
	≥12 m	6	4.17 (1.17)	2.17 (1.94)	−2.00	0.1041
	All	10	4.20 (1.87)	2.30 (1.83)	−1.90	0.0242
Abdominal pain (1−6)^b^, *mean (SD)*	<12 m	4	4.50 (1.29)	2.75 (1.26)	−1.75	0.1736
	≥12 m	6	2.67 (2.66)	1.17 (2.04)	−1.50	0.2500
	All	10	3.40 (2.32)	1.80 (1.87)	−1.60	0.0340
Bloating (1−6)^b^, *mean (SD)*	<12 m	4	3.75 (1.89)	3.00 (1.41)	−0.75	0.1489
	≥12 m	6	4.71 (1.11)	2.33 (1.86)	−2.17	0.0975
	All	10	4.36 (1.43)	2.60 (1.65)	−1.60	0.0213
Abdominal cramps (1−6)^b^, *mean (SD)*	<12 m	4	3.50 (1.00)	2.25 (1.50)	−1.25	0.1736
	≥12 m	6	2.71 (2.43)	0.50 (0.84)	−2.17	0.0975
	All	10	3.00 (2.00)	1.20 (1.40)	−1.80	0.0218
Vertigo (1−6)^b^, *mean (SD)*	<12 m	4	1.75 (1.50)	1.25 (1.89)	−0.50	1.0000
	≥12 m	6	1.29 (2.21)	0.50 (1.22)	−0.33	1.0000
	All	10	1.45 (1.92)	0.80 (1.48)	−0.40	0.5000
Spasms (1−6)^b^, *mean (SD)*	<12 m	3	3.67 (3.21)	3.33 (2.89)	−0.33	1.0000
	≥12 m	6	3.43 (1.99)	1.17 (1.33)	−1.83	0.0975
	All	9	3.50 (2.22)	1.89 (2.09)	−1.33	0.0568
Rectal bleeding (1−6)^b^, *mean (SD)*	<12 m	4	2.00 (2.45)	1.50 (2.38)	−0.50	1.0000
	≥12 m	6	0.50 (0.84)	0.00 (0.00)	−0.50	0.5000
	All	10	1.10 (1.73)	0.60 (1.58)	−0.50	0.1736

*F2−F1* change at treatment; *ICE* intermittent colonic exoperistalsis; *F1* feedback before treatment; *F2* feedback at treatment; *SD* standard deviation.

^a^Student’s *t*-test was used to compare quantitative variables.

^b^Wilcoxon matched-pairs test was used to compare semiquantitative variables.

Most symptoms of discomfort associated with chronic constipation were significantly reduced after treatment (Table 3). The strain to evacuate was reduced in 70.0% of participants, abdominal pain was reduced in 60.0% of participants, and bloating was reduced in 72.7% of participants. Abdominal cramps ameliorated in 72.7% of participants, while spasms improved in 60.0% of participants. When analyzed together, discomfort symptoms ameliorated in 56.1% of participants. Remarkably, five participants, of which four were under treatment for >12 months, reported suppression of chronic constipation symptoms while using MOWOOT. One reported suppression of all symptoms, which were reported at F1 with the maximum score (6, “Very severe”), except for vertigo, reported as “Absent.” Another participant-reported suppression of abdominal pain, spasms, and rectal bleeding; and, of the remaining three participants, one reported suppression of cramps and spasms, one reported rectal bleeding, and the third participant (under treatment for <12 months) reported vertigo.

**Table 3:** Discomfort symptoms, and laxatives and evacuation aids before (F1) and at treatment (F2) with the ICE device in-home care use.

		Treatment < 12 m *n* = 4		Treatment ≥ 12 m *n* = 7		All *n* = 11	
Discomfort symptoms		Yes (F1) /Same (F2)^a^	No (F1) /Better (F2)	Yes (F1) /Same (F2)^a^	No (F1) /Better (F2)	Yes (F1) /Same (F2)^a^	No (F1) /Better (F2)
Difficulty / strain	*F1*	3	1	6	0	9	1
	*F2*	0	4	2	4	2	8
	*OR*	21.00		23.40		36.00	
	*P*-value^b^	0.1429		0.0606		0.0055	
Abdominal pain	*F1*	4	0	4	2	8	2
	*F2*	1	3	1	5	2	8
	*OR*	21.00		10.00		16.00	
	*P-*value	0.1429		0.2424		0.0230	
Bloating	*F1*	4	0	7	0	11	0
	*F2*	1	3	2	4	3	7
	*OR*	21.00		27.00		49.29	
	*P-*value	0.1429		0.0210		0.0010	
Abdominal cramps	*F1*	4	0	5	2	9	2
	*F2*	1	3	0	6	1	9
	*OR*	21.00		28.60		40.50	
	*P-*value	0.1429		0.0210		0.0019	
Vertigo	*F1*	3	1	2	5	5	6
	*F2*	2	2	0	6	2	8
	*OR*	3.00		5.91		3.33	
	*P-*value	1.0000		0.4615		0.3615	
Spasms	*F1*	2	1	7	0	9	1
	*F2*	1	2	2	4	3	6
	*OR*	4.00		27.00		18.00	
	*P-*value	1.0000		0.021		0.0198	
Rectal bleeding	*F1*	2	2	2	4	4	6
	*F2*	1	3	0	6	1	9
	*OR*	3.00		7.22		6.00	
	*P-*value	1.0000		0.4545		0.3034	
Discomfort symptoms, overall^c^	*F1*	22	5	33	13	55	18
	*F2*	7	20	7	35	14	55
	*OR*	12.57		12.69		12.00	
	*P-*value	<0.0001		<0.0001		<0.0001	
Laxatives and evacuation aids		Yes (F1) /Same (F2)^d^	No (F1) /Less (F2)^d^	Yes (F1) /Same (F2)^d^	No (F1) /Less (F2)^d^	Yes (F1) /Same (F2)^d^	No (F1) /Less (F2)^d^
Laxatives	*F1*	3	1	7	0	10	1
	*F2*	3	1	6	1	9	2
	*OR*	1.00		3.46		2.22	
	*P*-value	1.0000		1.0000		1.0000	
Suppositories	*F1*	1	3	5	2	6	5
	*F2*	1	3	4	3	5	6
	*OR*	1.0000		1.87		1.40	
	*P-*value	1.0000		1.0000		1.0000	
Enemas/ Irrigation	*F1*	2	2	4	3	6	5
	*F2*	2	2	3	4	5	6
	*OR*	1.00		1.78		1.40	
	*P-*value	1.0000		1.0000		1.0000	
Digital stimulation	*F1*	2	2	3	4	5	6
	*F2*	2	2	3	4	5	6
	*OR*	1.00		1.00		1.00	
	*P-*value	1.0000		1.0000		1.0000	
Digital evacuation	*F1*	2	2	3	4	5	6
	*F2*	1	3	4	3	5	6
	*OR*	3.00		0.56		1.00	
	*P-*value	1.0000		1.0000		1.0000	
Total of laxatives and evacuation aids	*F1*	10	10	22	13	32	23
	*F2*	9	11	20	15	29	26
	*OR*	1.22		1.27		1.25	
	*P-*value	1.0000		0.8075		0.7014	

*ICE* intermittent colonic exoperistalsis; *F1* feedback before treatment; *F2* feedback at treatment; *OR* odds ratio.

^a^Worse discomfort symptoms was a possible option, but was not reported by any of the participants.

^b^All *p*-values were calculated using the Chi-squared/Fisher’s exact test.

^c^Total of discomfort symptoms analyzed together.

^d^“More” and “No use of evacuation aids” did not occur in any of the participants.

Analyses according to the time under treatment showed similar overall trends, with significant changes in participants under treatment for ≥12 months for bloating, abdominal cramps, and spasms but not for those under treatment for <12 months. However, changes in strain, abdominal pain, vertigo, and rectal bleeding were not statistically significant in any of the groups, whereas discomfort symptoms analyzed collectively significantly changed overall and in the two groups. Taken together, these results showed increased improvement in participants with ≥12 months vs. those with <12 months of use.

Regarding the use of laxatives and evacuation aids, three out of 11 participants reduced the use of laxatives, suppositories, or enemas (Table 3), although the difference before and after treatment did not reach statistical significance.

### Safety, usability, and treatment satisfaction

None of the participants reported any serious adverse events. Only one (9.1%) participant reported a sporadic episode of abdominal pain with spontaneous remission that did not require treatment interruption.

Changes in satisfaction with bowel function and management were statistically significant, with greater changes in the group treated for ≥12 months (Table 4). Remarkably, eight out of 11 participants reported being very satisfied/satisfied after treatment (*p* = 0.03). Regarding the evaluation of the ICE device (i.e., tolerability, efficacy, and ease of use), all participants rated tolerability within the highest categories, and most participants (8–9) did so for overall efficacy and ease of use of the ICE device (Table 5). Similarly, eight participants (of nine with available data) reported being very satisfied/satisfied in general with the ICE device (Table 5). Ratings were similar in the subgroups of participants according to time under treatment.

**Table 4:** Evaluation of satisfaction with bowel function and management before (F1) and at ICE treatment (F2).

Time under ICE treatment		<12 m (*n* = 4)		≥12 m (*n* = 7)		All (*n* = 11)	
*Mean (SD) scores*	F1	4.50 (1.73)		5.00 (1.41)		4.82 (1.47)	
	F2	2.75 (1.50)		2.71 (1.98)		2.73 (1.74)	
	F2 - F1	−1.75		−2.29		−2.09	
	*P-*value^a^	0.1018		0.0564		0.0095	
Score categories^**b**^		1−2	5−6	1−2	5−6	1−2	5−6
*Sample frequency (N)*	F1	1	3	1	6	2	9
	F2	3	1	5	2	8	3
	*P-*value^*c*^	0.4857		0.0406		0.0300	

*F2 - F1* change at treatment; *ICE* intermittent colonic exoperistalsis; *F1* feedback before treatment; *F2* feedback at treatment; *SD* standard deviation.

^a^Wilcoxon matched-pairs test.

^b^1, very satisfied; 2, satisfied; 5, rather dissatisfied; 6, dissatisfied.

^c^Chi-squared/Fisher’s exact test.

**Table 5:** Evaluation of tolerability, efficacy and usability of the ICE device at treatment (F2).

		Very good/Good	Satisfactory–Very insufficient	*P*-value^a^
		1−2	3−6	
Tolerability	<12 months	5	0	N/A
	≥12 months	6	0	
	All	11	0	
Overall efficacy	<12 months	3	2	0.5455
	≥12 months	5	1	
	All	8	3	
Ease of use	<12 months	3	2	0.1818
	≥12 months	6	0	
	All	9	2	
		Very satisfied/ Satisfied	Rather dissatisfied/ Dissatisfied	*P*-value
		1–2	5–6	
General satisfaction with the MOWOOT System	<12 months	3	0	N/A
	≥12 months	5	1	N/A
	All	8	1	N/A

*F2* feedback at treatment; *ICE* intermittent colonic exoperistalsis; *N/A* not applicable.

^a^Chi-squared/Fisher’s exact test for comparison between groups ( < 12 months vs. ≥12 months).

## Discussion

This structured patient feedback survey evaluated the effectiveness, tolerability, and satisfaction of the long-term in-home use of the ICE MOWOOT device in SCI individuals with chronic constipation. Participants reported a significant decrease in evacuation time and failed attempts to evacuate per week, along with an increased number of bowel movements after 10–24 months of ICE treatment. Discomfort symptoms of constipation were significantly reduced, and stool consistency softened with treatment despite the overall unchanged use of laxatives and evacuation aids, which are intended to soften stools, control evacuation, and prevent symptoms. Participants’ satisfaction with bowel function and management after device use increased and they rated its tolerability, efficacy, and ease of use as very good/good. Almost all participants were very satisfied/satisfied with the ICE device.

The MOWOOT device simulates the natural peristaltic contractions of the colon and can be used in the home setting without assistance. In the real-world setting of this survey, with no control over frequency and daily time of use, long-term in-home use of the ICE device provided a clinical benefit to SCI individuals. Importantly, the improved bowel function resulted in decreased time per evacuation. Even though no reduction in the use of evacuation aids was expected, feces showed a trend towards softer consistency while the use of evacuation aids tended to decrease. In this regard, almost all participants used laxatives before starting ICE treatment and, consequently, fecal consistency was generally optimal. Adding ICE device use to the bowel management routine slightly softened fecal consistency with no significant changes in the use of laxatives. In case excessive stool softening occurred in individuals combining ICE device use and laxatives, adjusting the dosage of laxatives would be suggested. Bowel dysfunction causes a profound impact on the lives of SCI individuals, limiting their daily activities and schedule due to the time needed per evacuation, among other reasons [15]. Even though assessing QoL was beyond the scope of this survey, the clinical benefits and reported satisfaction after ICE device use likely improved the QoL of SCI individuals.

Participant feedback was collected after at least ten months of regular ICE device use, capturing the benefits of long-term consistent use at home. Furthermore, the specific effects of prolonged use were assessed by comparing subgroups of participants according to time of device use (i.e., <12 months and ≥12 months). We used 12 months as the threshold for this analysis based on previous publications recommending a minimum study length of 6 months in long-term studies [16] and defining long-term follow-up from 12 months and onwards in studies assessing the effects of TAI in individuals with NBD, such as SCI [17]. Bowel function improved in both groups, although to a greater extent in the long-term ( ≥ 12 months) group, indicating overall increased benefits in participants who used the ICE device longer. Likewise, discomfort symptoms decreased in both groups, but participants who used the ICE device for ≥12 months reported significantly decreased bloating, cramps, and spasms. Even though, due to the small sample size, changes lacked statistical significance across subgroups and variables, the differences observed in both groups were likely clinically significant in terms of positive impact on QoL, supporting the long-term use of the ICE device. Altogether, these results suggest that a regular, prolonged use of the ICE device may provide greater improvement in bowel function and chronic constipation symptoms.

A recent clinical trial has reported the benefits of ICE treatment in patients with chronic constipation [13]. This trial showed reduced symptoms of discomfort and reduced use of evacuation aids, demonstrating the efficacy of a four-week intervention. Similar results regarding constipation symptoms were found in our real-world survey, evaluating participant-reported benefits after at least ten months of in-home use, under no supervision by healthcare professionals. Compared with the four-week intervention in different patient profiles regarding the etiology of constipation, this study’s results suggest that SCI individuals require a prolonged use to obtain clinical benefits. Additionally, the number of adverse events was low in both studies, consistent with the favorable safety of the treatment, and both studies reported a significant increase in satisfaction after treatment. Furthermore, in our survey, participants rated tolerability, efficacy, and ease of use highly; and an increased satisfaction reflected the effectiveness of its long-term use without adverse events. In this regard, satisfaction with bowel function and management correlates with increased QoL [18], further supporting the inclusion of the ICE device in the bowel routine of SCI individuals.

Professional manual abdominal massage has been described as an effective method to treat chronic constipation due to different etiologies [12, 19, 20], including SCI [21, 22]. In SCI individuals, manual massage has positive effects on clinical symptoms, such as a higher frequency of defecation and minor fecal incontinence and abdominal distension, similar to this survey assessing the use of the ICE device [22]. Manual massage requires a therapist for its daily application, which is unfeasible and expensive for patients on the healthcare system. Therefore, ICE treatment constitutes a feasible, cheaper alternative to manual abdominal massage, and it’s accessible to more individuals. Importantly, it may even be more effective owing to the regularity of treatments.

Another method to treat chronic constipation is TAI [17, 23]. This technique, which stimulates evacuation, is minimally invasive, recommended in case of failure of conservative methods, and its use has also been reported in individuals with SCI [5, 24, 25]. TAI can be self-administered after specific training, reducing discomfort and clinical symptoms associated with chronic constipation [9]. Similar to our survey showing a 24.5-min reduction in time per evacuation after the ICE device use, a previous observational study assessing the efficacy and safety of a TAI device in SCI individuals with chronic constipation reported reduced time spent on their daily bowel management by 27.5 min [26]. Even though self-perceived severity of constipation worsens significantly over time in SCI individuals [27], the reported abandon rate for TAI treatment at 21 months in SCI individuals was 38% [28], whereas adherence to ICE treatment in previous studies and in this survey, particularly after >12 months of use, was >95% [13]. Furthermore, long-term studies assessing TAI only showed a 35% success rate after three years in patients with NBD [29]. In this study, daily time of device use exceeded the recommended length, yielding mean adherence rates >100% regardless of the total period of use.

The results from this survey should be interpreted in the context of limitations associated with sample size. Despite this limitation, results in the whole study sample were robust. Likewise, while the limited sample size may have decreased the statistical power of the subgroup analysis, some variables still yielded statistically significant results and, overall, supported increased benefits of longer treatments. Future larger studies assessing the long-term use of the ICE device may be needed to confirm these observations. The study population was not stratified according to SCI-upper vs. SCI-lower motor neuron dysfunction, which could be considered a limitation. However, this study, rather than focusing on NBD, is an evaluation of SCI individuals with chronic constipation, one of the symptoms of NBD, in a real-life situation. MOWOOT simulates the natural peristaltic contractions of the colon, like manual colonic massage, which is the recommended therapy for both upper and lower motor neuron dysfunctions in individuals with SCI [5]. This survey has a series of strengths related to its real-world setting, without strict selection criteria and monitoring or specific control over frequency and time of use, capturing the benefits of in-home use in the relevant survey setting. Good safety and effectiveness profiles shown in this survey and previous studies [13], together with high adherence to treatment, suggest that the ICE device will be effective to treat chronic constipation in the home setting regardless of the patient profile in terms of the underlying chronic constipation etiology.

In conclusion, long-term in-home ICE treatment improved bowel function and symptoms of chronic constipation in individuals with SCI, providing clinical benefits to this population. Due to the feasibility of its use in the home setting and participant-reported excellent adherence, the ICE device may be a good option to treat chronic constipation. Integrating the ICE device use to the bowel management routine may positively impact SCI individuals’ health, likely contributing to improving their QoL as well.

## Supplementary information

Supplementary Material

Check list

## Data Availability

The datasets generated and/or analyzed during this study are available from the corresponding author on reasonable request.
