# Supplementary material for: Intermittent colonic exoperistalsis for chronic constipation in spinal cord-injured individuals. A long-term structured patient feedback survey to evaluate home care use
Source: Spinal Cord Ser Cases. 2023 Jul 29;9:37. doi: 10.1038/s41394-023-00597-z (PMC10387045; doi:10.1038/s41394-023-00597-z)
Supplement: Supplementary file 1 — Supplementary Material [file 41394_2023_597_MOESM1_ESM.pdf]

Supplementary Material for the Submission by Herrero-Fresneda et al. of "Intermittent Colonic Exoperistalsis for Chronic Constipation in Spinal Cord-Injured Individuals. A Long-term Structured Patient Feedback Survey to Evaluate Home Care Use."

Supplementary Methods

Variables and Assessments

At F2 (TREAT), participants reported on device use as number of sessions/day, session duration (min), and the total period of MOWOOT use (months) until F2 (participants may have continued treatment with the ICE device thereafter). Adherence to treatment was defined as the percentage of minutes of use per day over 20 min, the maximum time of daily use instructed.

Bowel function effectiveness was assessed by number of bowel movements/week, number of failed attempts to evacuate/week, number of incomplete bowel movements/week, and time (min) spent per evacuation. The semiquantitative Bristol stool scale [1] was used to evaluate fecal consistency on a 1 (hard feces) to 7 (liquid diarrhea) range. Effectiveness variables (semiquantitative and qualitative) related to symptoms of constipation were difficulty/pressing (strain), abdominal pain, bloating, abdominal cramps, vertigo, spasms, and rectal bleeding. Participants were asked to rate symptoms on a 0–6 scale, from "No symptoms" to "Very severe symptoms." Additionally, based on ratings and changes before and after treatment, the answers per time point were categorized as "Yes" and "No" at F1 (PRE); and as "Worse," "Same," "Better," or "No symptoms" at F2 (TREAT).

The concomitant use of laxatives (type and dose) and evacuation aids—intended to control evacuation and prevent accidents—, including suppositories (type and dose), enemas/irrigation, digital stimulation, and digital evacuation, were also collected. Their use was categorized as "Yes" or "No" at F1 (PRE) and, when applicable, as "More," "Same," "Less," or "No" at F2 (TREAT). Participants reported satisfaction with bowel function and management at F1 (PRE) and at F2 (TREAT) as "Very satisfied," "Satisfied," "Rather dissatisfied," and "Dissatisfied," and scores of 1, 2, 5, and 6, respectively, were assigned.

Participants evaluated the efficacy, tolerability/side effects, and ease of use on a 1–6 scale, ranging from “Very good” (1) to “Very insufficient” (6), and rated general satisfaction with the MOWOOT system as “Very satisfied” (1), “Satisfied” (2), “Rather dissatisfied” (5), and “Dissatisfied” (6) at F2 only. Participants reported adverse events and their severity. Finally, they were asked whether their drinking or eating habits had changed significantly during ICE treatment (“Yes/No”) and explain how (free text). The investigator collecting and analyzing the data was blinded to participants’ profiles (such as demographic characteristics and treatment time).

The translated version (German to English) of the survey questionnaire is included below.

Statistical Analysis

Paired measures were compared using the Student’s t-test for quantitative variables and the non-parametric Wilcoxon test for semiquantitative variables. The number of daily ICE device sessions and their duration was compared between subgroups of participants using the two-way ANOVA. The effect sizes of the differences before treatment (F1) and at the second feedback (F2) were analyzed using odds ratios (ORs) and the Chi-squared/Fisher's exact test using contingency tables.

References

[1] Lewis SJ, Heaton KW. Stool form scale as a useful guide to intestinal transit time. Scand J Gastroenterol 1997;32. https://doi.org/10.3109/00365529709011203.

Long Term Use of the MOWOOT System for Chronic Constipation

Please tick where applicable and complete the empty fields.

	**Earlier,**<br>about a year ago without MOWOOT	**Now,**<br>after about a year of using MOWOOT
How often were you able to empty your bowels per week?	______ times per week	______ times per week
How much time did it take you on average to empty your bowels, including preparation?	________ min.	________ min.
Unsuccessful bowel evacuations:	______ times per week	______ times per week
Incomplete bowel evacuations:	______ times per week	______ times per week
Average stool consistency (Bristol scale 1 - 7)	______	______
What complaints did you experience?	Please rate "0" if the following complaints did not occur in the past or no longer occur. If the complaints did occur in the past or do occur now, indicate a 1 for very mild complaints and ascending to 6 for very severe complaints.	
- Difficult defecation/pressing:	☐ 0<br>☐ 1 ☐ 2 ☐ 3 ☐ 4 ☐ 5 ☐ 6	☐ 0<br>☐ 1 ☐ 2 ☐ 3 ☐ 4 ☐ 5 ☐ 6
- Pain	☐ 0<br>☐ 1 ☐ 2 ☐ 3 ☐ 4 ☐ 5 ☐ 6	☐ 0<br>☐ 1 ☐ 2 ☐ 3 ☐ 4 ☐ 5 ☐ 6
- Bloating	☐ 0<br>☐ 1 ☐ 2 ☐ 3 ☐ 4 ☐ 5 ☐ 6	☐ 0<br>☐ 1 ☐ 2 ☐ 3 ☐ 4 ☐ 5 ☐ 6
- Abdominal cramps	☐ 0<br>☐ 1 ☐ 2 ☐ 3 ☐ 4 ☐ 5 ☐ 6	☐ 0<br>☐ 1 ☐ 2 ☐ 3 ☐ 4 ☐ 5 ☐ 6
- Vertigo	☐ 0<br>☐ 1 ☐ 2 ☐ 3 ☐ 4 ☐ 5 ☐ 6	☐ 0<br>☐ 1 ☐ 2 ☐ 3 ☐ 4 ☐ 5 ☐ 6
- Spasticity	☐ 0<br>☐ 1 ☐ 2 ☐ 3 ☐ 4 ☐ 5 ☐ 6	☐ 0<br>☐ 1 ☐ 2 ☐ 3 ☐ 4 ☐ 5 ☐ 6
- Rectal bleeding	☐ 0<br>☐ 1 ☐ 2 ☐ 3 ☐ 4 ☐ 5 ☐ 6	☐ 0<br>☐ 1 ☐ 2 ☐ 3 ☐ 4 ☐ 5 ☐ 6
Which therapy measures were/are carried out?		
- Oral laxative (name /daily dose):		
- Suppositories, enemas (name /daily dose):		
- other	☐ Digital Stimulation<br>☐ Digital Evacuatiom<br>☐ Enema / Irrigation	☐ Digital Stimulation<br>☐ Digital Evacuatiom<br>☐ Enema / Irrigation
How satisfied were/are you with the treatment of your constipation overall?	☐ very satisfied<br>☐ satisfied<br>☐ rather dissatisfied<br>☐ dissatisfied	☐ very satisfied<br>☐ satisfied<br>☐ rather dissatisfied<br>☐ dissatisfied
	Explanation:	Explanation:

Personal details:

☐ male ☐ female

Age:

☐ Under 20, ☐ 20 to 29, ☐ 30 to 39, ☐ 40 to 49, ☐ 50 to 59, ☐ 60 to 69, ☐ over 70 years old

Reason for MOWOOT treatment:

☐ Chronic constipation associated with neurogenic bowel (spinal cord damage)

☐ Chronic constipation due to

____

Use of the MOWOOT-System:

Duration: _______ months

Frequency: ☐ twice per day ☐ daily ☐ every ___ day

Duration per application: _____ Minutes Level _____

Has your drinking or eating behaviour changed significantly during this time? ☐ yes

☐ no

If yes,
how?____

Evaluation of the MOWOOT system in long-term use.

How would you rate the following MOWOOT characteristics?
1 = very good, 2 = good, 3 = satisfactory, 4 = sufficient, 5 = poor, 6 = very insufficient.

MOWOOT–	Rating	Explanation:
Effect / Efficacy	____	
Tolerability / Side Effects	____	
Ease of Use	____	

How satisfied are you with the MOWOOT system overall?

☐ very satisfied ☐ satisfied ☐ rather dissatisfied ☐ dissatisfied

Explanation:

Would you recommend MOWOOT to other "fellow sufferers"?

☐ very likely ☐ likely ☐ rather unlikely ☐ unlikely

And finally, how would you rate the service provided by 4M Medical GmbH?
1 = very good, 2 = good, 3 = satisfactory, 4 = sufficient, 5 = poor, 6 = very insufficient.

	Rating	Explanation:
Support by the 4M Service	____	

Space for your comments:

Thank you for your participation!
